# The Use of High-Flow Nasal Cannula in the Emergency Department and a Comparison of Its Efficacy With Noninvasive Ventilation

**DOI:** 10.7759/cureus.65709

**Published:** 2024-07-29

**Authors:** Varsha Shinde, Sharmila J Mavudelli

**Affiliations:** 1 Department of Emergency Medicine, Dr. D. Y. Patil Medical College, Hospital and Research Centre, Pune, IND

**Keywords:** high-flow nasal cannula, high-flow nasal oxygenation, noninvasive ventilation, acute dyspnea, acute hypercapnic failure, acute hypoxemic respiratory failure

## Abstract

Background: High-flow nasal cannula (HFNC) oxygenation has emerged as a convenient and handy oxygenation mode over the past few years, especially during the COVID-19 pandemic. HFNC is designed to provide humidified oxygen at high flow rates to subjects in a much more patient-compliant method. Noninvasive ventilation (NIV) has been a powerful tool in treating dyspneic patients of different etiologies, yielding positive outcomes over many decades. HFNC has the potential to serve as an alternative to NIV for acutely breathless patients, offering better patient compliance.

Methods: A prospective observational study was conducted with a population size of 100 patients. The patients were randomly assigned to HFNC and NIV groups and further compared based on the clinical criteria, arterial oxygen pressure (PaO_2_)/fraction of inspired oxygen (FiO_2_) ratios, and modified Borg score. Simple proportions, mean, standard deviation, and chi-square tests were used. The chi-square test was applied to determine the association between the two attributes.

Results: Both HFNC and NIV subset populations have shown substantial improvement in their clinical criteria in terms of respiratory rate, heart rate, oxygen saturation, PaO_2_/FiO_2_ ratios, and modified Borg score over two and six hours with statistically significant improvement in oxygen saturations among HFNC subset in comparison to NIV subset (at two hours, p = 0.004; at six hours, p = 0.022). Secondary outcomes like the need for intubation (14% in HFNC, 22% in NIV) and mortality (4% in HFNC, 6% in NIV group) were noted, which were statistically insignificant in comparing their efficacy.

Conclusion: The study concluded that HFNC resulted in better clinical parameters than NIV, but the difference was statistically insignificant except for oxygen saturation. Similarly, HFNC resulted in a decreased need for intubation and less mortality compared to NIV.

## Introduction

Dyspnea is one of the most common problems encountered in patients presenting to the emergency department (ED) and is defined as discomfort in breathing [1]. Dyspnea might differ in intensity and characteristics based on the etiology, and patients perceive it differently based on their social, cultural, and psychological backgrounds [2]. Respiratory failure occurs when the lungs fail to maintain normal arterial oxygen and carbon dioxide levels [1]. It can be further classified into type 1 and type 2 respiratory failures. Type 1 respiratory failure is characterized by acute hypoxemic failure, while type 2 is characterized by acute hypoxemic failure with hypercapnia. The intensity of dyspnea at ED presentation further determines the need for hospital admission [3]. It is, in fact, analogous to the duration of hospital stay along with in-hospital prognosis and mortality [4,5]. This poses a challenging task to the ED physician as they must reach a rapid diagnosis with the limited clinical information available and further initiate a treatment plan to stabilize the patient [6].

Conventional oxygen therapy (COT), like a simple nasal cannula, Hudson’s face mask, and nonrebreather mask, can be used in treating acute dyspnea in ED [7]. While a number of noninvasive oxygen delivery devices are available to the ED physician’s aid, each has its limitations. Nasal cannulas and face masks, while having the advantage of good patient compliance, face the limitations of a delivered fraction of inspired oxygen (FiO_2_). An extremely hypoxemic patient cannot be well supported on these devices, leading to eventual therapeutic failure. A high-flow nasal cannula (HFNC), as discussed earlier, is now being used in EDs for managing acute dyspnea patients [8-11].

Noninvasive ventilation (NIV) has proven itself beyond reasonable doubt as a lifesaving modality in hypoxemic respiratory failure secondary to various etiologies like chronic obstructive pulmonary disease (COPD) and acute heart failure. Poor patient compliance, risk of aspiration, and leakage leading to compromised FiO_2_ are just some of the factors that may hinder its usage. The eventuality that one is trying to avoid in such clinical scenarios is endotracheal intubation. Hence, an ED physician could definitely use a potent, noninvasive oxygen delivery device with fewer complications and limitations.

From this need, we have diverted our focus to the untapped therapeutic potential of HFNC. HFNC device is designed to deliver a humified air-oxygen blend through a nasal cannula to deliver flow rates as high as 60 L/minutes. It can provide the desired FiO_2_ ranging from 0.21 to 1, which can be adjusted according to the patient’s needs. HFNC also helps prove positive end-expiratory pressure (PEEP) and dead space washout [12-15]. The patient can still perform their regular activities like eating, speaking, and drinking, which makes HFNC more tolerable by patients [16,17]. However, HFNC is a noninvasive mode of oxygenation, while NIV is a noninvasive mode of ventilation. While there is a dearth of data on the subject, the available literature has displayed extremely encouraging results. From a research point of view, while it may seem to be uncharted waters, we feel the need to tread into this territory would lead to a generation of an extremely useful pool of data, which not only would fill the academic void that is there concerning HFNC but would also benefit a large number of patients visiting our ED.

The focus of the study is going to be the patients rushing to the ED with breathlessness and in requirement of noninvasive means of oxygen delivery. The varying etiologies that would be covered under this spectrum would be COPD, bronchial asthma, acute respiratory distress syndrome, and acute decompensated heart failure, to name a few. It has been noted that there has been a lot of discrepancy and controversy regarding the role and efficacy of HFNC in treating dyspneic patients with respect to other available modalities of noninvasive oxygenation and NIV. Thus, this study aims to compare the use of HFNC and NIV in managing acutely dyspneic subjects with different etiologies in the emergency department.

## Materials and methods

Study design

A prospective observational study was conducted in the Department of Emergency Medicine of Dr. D. Y. Patil Medical College, Hospital and Research Centre, Pimpri, Pune, to compare the efficacy of HFNC with NIV in patients presenting with acute dyspnea to ED in terms of improvement in clinical parameters, modified Borg's scale, and adverse effects. The study was conducted over a period of one year, i.e., June 2023 to May 2024. A sample size of 100 was selected based on convenience, with a 1:1 ratio in each group, i.e., 50 patients each in the NIV and HFNC groups. Certain criteria were set for the selection of the study population. Inclusion criteria include all patients admitted to ED with dyspnea requiring either HFNC or NIV as the modality of treatment who have given informed consent for the study. Patients requiring other modalities of oxygenation and ventilation, i.e., nasal prongs, face masks, nonrebreather masks, and invasive ventilation, were excluded. The study was further approved by the Institutional Ethical Committee.

Devices

Optiflow Adult (HFNC device) manufactured by Fisher and Paykel Healthcare, Auckland, New Zealand, which delivers humidified air mixed with oxygen at high flow rates of up to 60 L/minutes with FiO_2_ ranging from 0.21 to 1, was used for the study, which delivered the humidified air through nasal cannulas. Maquet Servo S version 8.0 (NIV device; Maquet, Rastatt, Germany) was used for NIV. The device had both NIV and invasive ventilation modes. The delivered PEEP could be set, and further FiO_2_ can be adjusted as per the patient's requirement from 0.21 to 1.

Study protocol

All the patients matching the inclusion criteria were registered for the study. Upon arrival, the patient’s demographic data, clinical history, vitals, including heart rate (HR), blood pressure (BP), oxygen saturation (SpO_2_), and respiratory rate (RR), and modified Borg scale [18] for dyspnea were noted. The modified Borg scale is a numerical scaling of the patient's perception of the severity of breathlessness ranging from 0 to 10. A detailed clinical examination was done. An arterial blood gas was performed, and arterial oxygen pressure (PaO_2_)/FiO_2_ ratios were calculated and recorded. Patients were then randomly designated into HFNC and NIV subsets based on equipment availability. In the NIV group, patients were taken on pressure support and pressure control modes based on their breathing work and compliance with a set PEEP and FiO_2_ values, and the subject was further observed at the end of two and six hours for clinical improvement. Similarly, the patients in the HFNC group were started with minimum flow rates and FiO_2_ and further titrated up as per the patient's needs and further observed for improvement at two and six hours. The two subsets were then analyzed for primary outcomes, which included improvement in clinical parameters, including HR, SpO_2_, BP, RR, and PaO_2_/FiO_2_ ratio, and a modified Borg scale. The secondary outcomes included cost-benefit (requirement of intubation) and adverse effects (mortality).

Statistical analysis

The sample size was selected by convenience to be 100. The data were tabulated in a Microsoft Excel sheet (Microsoft Corporation, Redmond, WA) and analyzed with the help of IBM SPSS software version 28 (IBM Corp., Armonk, NY). Simple proportions, mean, and standard deviation were used for the numerical data, and further, they were subjected to a test. The chi-square test was used for categorical data. The chi-square test was applied to determine the association between the two attributes. A p value of less than 0.05 was taken as statistically significant. A post-test hoc analysis was done using WinPepi software version 11.65 (updated by J. H. Abramson) to determine the power of the study.

## Results

The study included a total population of 100 patients. Subjects presenting to ED with dyspnea were included in the study. Among the 100 people, 41 were female, and 59 were male. Patients presenting with multiple etiologies for dyspnea were observed in the study conducted, and the wide range application of HFNC in patients presenting to ED with different etiologies was recorded (Table 1). Among the HFNC group, the most common etiologies were identified to be lower respiratory tract infections (LRTIs; 42%), followed by heart failure (18%) and septic shock (10%), whereas in the NIV group, the most common etiologies included LRTI (34%) and fluid overload in chronic kidney disease (20%), followed by heart failure (14%).

**Table 1:** Etiologies of the study population HFNC: high-flow nasal cannula; NIV: noninvasive ventilation; COPD: chronic obstructive pulmonary disease; OAD: obstructive airway disease; LRTI: lower respiratory tract infection; OSA: obstructive sleep apnea; GI: gastrointestinal

Diagnosis	HFNC	NIV	Grand total
Abdominal sepsis	1 (2%)	0 (0%)	1 (1%)
Cardiogenic shock	0 (0%)	1 (2%)	1 (1%)
COPD exacerbation	0 (0%)	2 (4%)	2 (2%)
Corrosive poisoning	1 (2%)	0 (0%)	1 (1%)
Diabetic ketoacidosis	0 (0%)	2 (4%)	2 (2%)
Fluid overload	4 (8%)	10 (20%)	14 (14%)
Heart failure	9 (18%)	7 (14%)	16 (16%)
Hepatorenal syndrome	1 (2%)	0 (0%)	1 (1%)
Infective exacerbation of OAD	1 (2%)	1 (2%)	2 (2%)
LRTI	21 (42%)	17 (34%)	38 (38%)
Lung fibrosis	1 (2%)	0 (0%)	1 (1%)
Myocardial infarction	1 (2%)	2 (4%)	3 (3%)
OSA with type 2 respiratory failure	0 (0%)	1 (2%)	1 (1%)
Pleural effusion	0 (0%)	1 (2%)	1 (1%)
Pneumothorax	1 (2%)	0 (0%)	1 (1%)
Pulmonary embolism	2 (4%)	1 (2%)	3 (3%)
Pulmonary Koch	0 (0%)	2 (4%)	2 (2%)
Seizure	1 (2%)	0 (0%)	1 (1%)
Septic shock	5 (10%)	2 (4%)	7 (7%)
Trauma	1 (2%)	0 (0%)	1 (1%)
Upper GI bleed	0 (0%)	1 (2%)	1 (1%)
Total	50 (100%)	50 (100%)	100 (100%)

**Figure 1:**
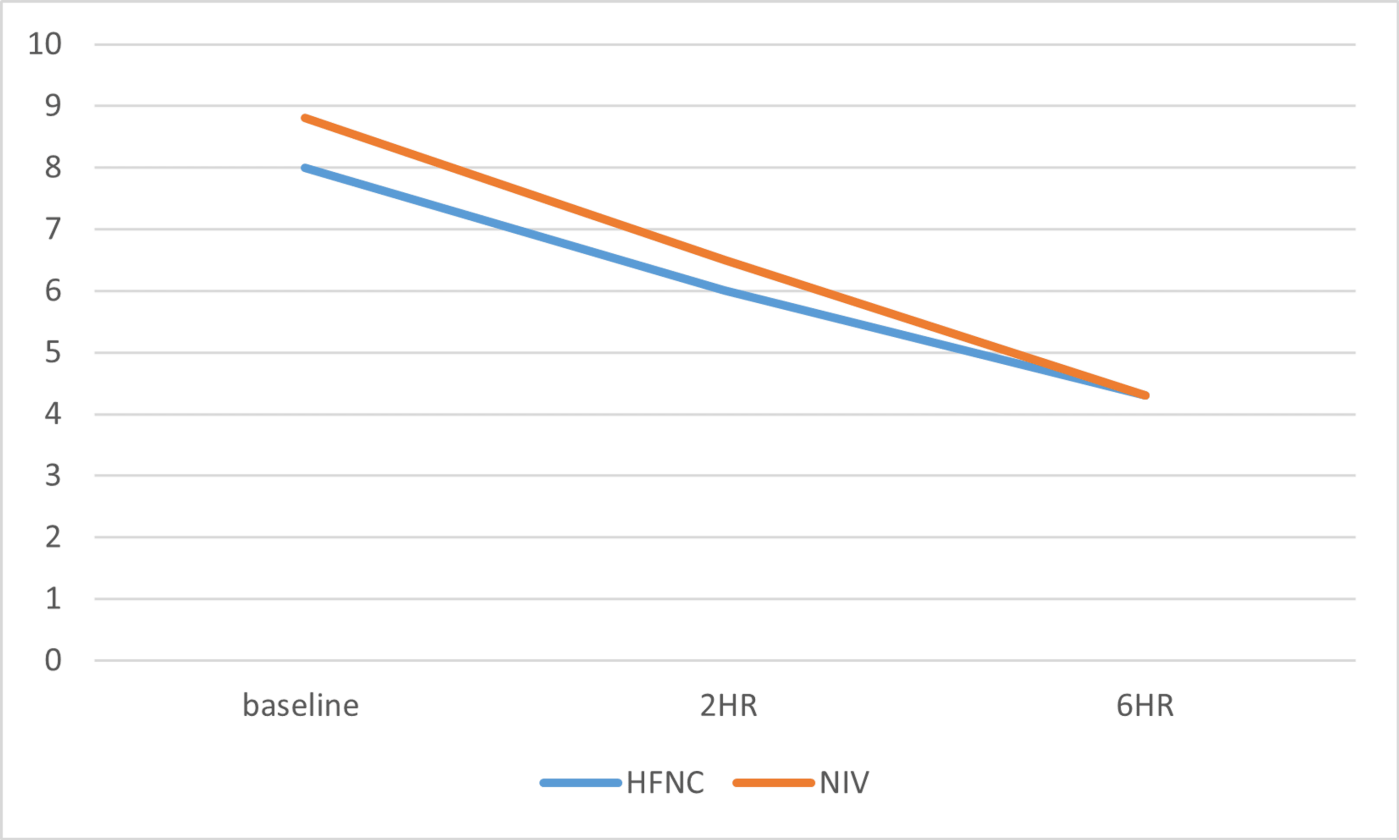
Mean modified Borg scores of the subjects over the observation period Sample size (n) = 100; the mean, standard deviation, and standard errors of the data were calculated and further subjected to unpaired t-tests among the NIV and HFNC groups. The p values at baseline, two hours, and six hours were 0.003, 0.25, and 0.911, respectively HFNC: high-flow nasal cannula; NIV: noninvasive ventilation

During the six-hour observation period, both groups were monitored, and various clinical parameters were compared between them at the two- and six-hour marks using unpaired t-tests. The modified Borg scale, a subjective parameter, showed no statistically significant improvement between the NIV and HFNC groups at two hours (p = 0.25) and six hours (p = 0.911) (Figure 1).

**Figure 2:**
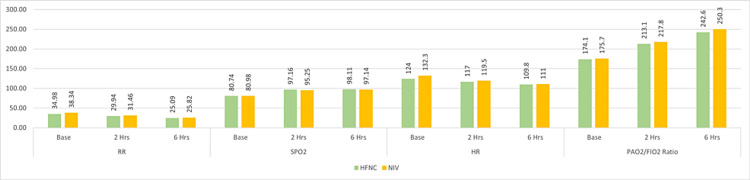
Clinical parameters of the population The clinical parameter mean values among the NIV and HFNC groups are subjected to unpaired t-test. Sample size N =100; RR exhibited statistically significant changes, with a p value of 0.013 at baseline, 0.269 at two hours, and 0.491 at six hours. Similarly, SpO_2_ levels varied significantly, registering p values of 0.897 at baseline, 0.004 at two hours, and 0.022 at six hours. HR also displayed notable differences, with p values of 0.025 at baseline, 0.456 at two hours, and 0.705 at six hours. In contrast, the PaO_2_/FiO_2_ ratio did not show statistically significant changes, recording p values of 0.869 at baseline, 0.675 at two hours, and 0.471 at six hours RR: respiratory rate (breaths/minutes); SpO_2_: oxygen saturation (%); HR: heart rate (beats/minutes); PaO_2_: partial pressure of oxygen; FiO_2_: fraction of inspired oxygen; HFNC: high-flow nasal cannula; NIV: noninvasive ventilation

The HFNC group exhibited mean RRs of 35/minute, 30/minute, and 25/minute at baseline, two hours, and six hours after intervention, indicating notable improvement (Figure 2). Nevertheless, compared to the NIV group, these results did not reach statistical significance at two hours (p = 0.269) and six hours (p = 0.49). The mean SpO_2_ in the HFNC group improved from 81% at baseline to 97% at two hours and 98% at six hours (Figure 2), showing substantial enhancement. Statistical analysis revealed significantly better saturation levels in the HFNC group compared to the NIV group at two hours (p = 0.004) and six hours (p = 0.022). Regarding HR, the patients in the HFNC group exhibited mean values of 124, 117, and 110 bpm at baseline, two hours, and six hours, respectively, indicating a significant decrease over time (Figure 2). However, there were no statistically significant differences between the HFNC and NIV groups at two hours (p = 0.456) and six hours (p = 0.705). The PaO_2_/FiO_2_ ratio, derived from arterial blood gas analysis, demonstrated notable improvement in the mean values for the HFNC group: 174, 213, and 242 at baseline, two hours, and six hours, respectively (Figure 2). However, there were no significant differences between the NIV and HFNC groups at two hours (p = 0.675) and six hours (p = 0.471). These findings underscore varied responses in clinical parameters between the HFNC and NIV groups over the observed time intervals.

Other important categorical parameters compared between the groups included the need for intubation and mortality in the ED. The results were subjected to a chi-square test to determine if they were statistically significant. Of the 50 members in the HFNC group, seven patients needed intubation within six hours, whereas 11 patients in the NIV group needed intubation, but the difference between the two groups is statistically insignificant (p = 0.298). Mortality in the ED was comparatively higher in the NIV group (3 in 50) than in the HFNC group (2 in 50), but the difference was statistically insignificant. The post-test hoc analysis confirmed that the study was adequately powered to detect the mean deviation in SpO_2_ between the two modalities, i.e., HFNC and NIV at two hours (power = 83.24%) but inadequately powered for six hours (67.65%) and other outcomes.

## Discussion

To the best of our knowledge, the present study is the first among the research studies done in the Indian ED background to compare HFNC with NIV in acute dyspneic patients. The final outcomes of the study depicted that HFNC significantly improved respiratory distress in dyspneic patients and improved patient compliance compared to NIV. Rittayamai et al. documented the proficiency of HFNC in the treatment of acute dyspnea and hypoxia in 40 subjects who presented to the ED. They concluded that the patient was symptomatically better after one hour of HFNC [19]. The present study has shown the efficacy of HFNC with regard to improvement in clinical parameters, PaO_2_/FiO_2_ ratios, and modified Borg scores in the study population over a period of six hours. The efficacy of HFNC can be attributed to its working principle, which supplies humidified oxygen with a capacity to give higher flow rates, which allows the washout of pharyngeal dead space. It also helps increase the functional residual capacity of the lungs by giving PEEP.

Makdee et al. carried out a randomized controlled trial (RCT), which included 128 subjects, to compare the efficacy of HFNC against COT in treating cardiogenic pulmonary edema and concluded that, at the end of the first treatment, HFNC resulted in significant improvement in respiratory distress in cardiogenic pulmonary edema patients [20]. Zhao et al. conducted a systematic review and meta-analysis including 11 RCTs, eight comparing HFNC vs. COT, two comparing NIV vs. HFNC, and one comparing COT, NIV, and HFNC from data acquired from different intensive care settings. They concluded that compared to COT, the population subjected to HFNC had a decreased need for intubation and ventilation. HFNC did not have superior outcomes when compared to NIV. However, this study was conducted in an ICU setting where the patient is shifted after being stabilized in the ED [21,22]. The present study outcomes show that more people in the NIV subset needed intubation in contrast to the HFNC subset, and adverse outcomes like death were more in the NIV group. The clinical parameters had significantly improved in both NIV and HFNC groups, but only improvement in SpO_2_ was statistically significant, whereas HFNC had a better result. Huang et al. conducted a meta-analysis study that included 775 subjects regarding using HFNO vs. COT in acute respiratory failure patients presenting to ED. They concluded that treating acute renal failure patients in EDs with HFNC might decrease the intubation rate compared to COT. It can also alleviate the need for escalation, decrease the patient's dyspnea severity, and improve the patient’s comfort compared to COT [23]. Tinelli et al., in their meta-analysis and systematic review of 775 subjects, compared HFNC vs. NIV and COT and concluded that HFNC had no benefits compared with COT and NIV in terms of the need for endotracheal intubation, failure of treatment, length of hospital stay, and fatality; COT had better patient compliance [24]. Koga et al. studied the various applications of HFNC vs. NIV and concluded that HFNC was more beneficial to nonhypercapnic and LRTI patients than hypercapnic distress. Our study has shown that HFNC improves clinical outcomes in LRTI and heart failure patients. However, HFNC has its limitations in hypercapnic failure patients; thus, large multicentric randomized trials are needed to understand this aspect better [25]. Marjanovic et al. worked on a systematic review to study the efficacy of COT against HFNO in 673 patients of acute respiratory failure and concluded that the early initiation of HFNO in acute respiratory failure patients did not show any reduction in intubation requirement in comparison to COT [26]. Respiratory efforts and rates improved in the HFNC group. Ovtcharenko et al. published a study of multiple RCTs conducted to study HFNC vs. NIV therapy in type 2 respiratory failure subjects and concluded that there is limited evidence for establishing the efficacy of HFNC in comparison to NIV in the management of patients with acute hypercapnic respiratory failure [27-29]. Nair et al. conducted a study to assess the outcomes, such as mortality and hospital length of stay, in COVID-19 pneumonia patients requiring either invasive or NIV. Their findings indicated that having preexisting respiratory conditions was associated with worse outcomes [30].

Similar to other research endeavors, this study has its own set of limitations. It compares HFNC, a method of oxygenation, with NIV, which operates on different mechanistic principles. Additionally, the study population was randomly divided into groups without specific criteria, potentially affecting the precision of results due to variations in initial patient severity upon ED admission, a crucial determinant of prognosis. Limited detail is provided regarding the application of HFNC in type 2 respiratory failure. The study was found to be adequately powered to detect mean deviation in SpO_2_ between the two modalities, i.e., HFNC and NIV at two hours, but was underpowered for other outcomes.

## Conclusions

The study found that HFNC yielded similar clinical outcomes to NIV, although the differences were statistically nonsignificant except for SpO_2_. Likewise, HFNC demonstrated reduced intubation requirements and lower mortality rates compared to NIV, although these findings did not reach statistical significance. HFNC has shown efficacy in improving patient outcomes by reducing respiratory effort and enhancing patient compliance. Nonetheless, larger randomized multicenter studies are needed to establish robust correlations.
